# Is the treatment of inflammatory arthritis different in sickle cell disease?

**DOI:** 10.55730/1300-0144.5515

**Published:** 2022-07-13

**Authors:** Gezmiş KİMYON, Gül İLHAN

**Affiliations:** 1 Division of Rheumatology, Department of Internal Medicine, Faculty of Medicine, Hatay Mustafa Kemal University, Hatay, Turkey; 2 Division of Hematology, Department of Internal Medicine, Faculty of Medicine, Hatay Mustafa Kemal University, Hatay, Turkey

**Keywords:** Arthritis, sickle cell disease, steroid, methotrexate, anti-TNF

## Abstract

**Background/aim:**

Musculoskeletal findings are common in sickle cell patients and may be confused with inflammatory arthritis. In addition, complications such as frequent infections may create difficulties while choosing drugs such as steroids, methotrexate, or anti-TNFs. Our aim is to reveal whether the treatment is different in sickle cell patients with rheumatic diseases such as rheumatoid arthritis.

**Materials and methods:**

Patients followed by Rheumatology and Hematology divisions of Hatay Mustafa Kemal University Hospital were retrospectively screened. Excluding patients with musculoskeletal findings associated with sickle cell disease (SCD), patients with chronic or acute inflammatory arthritis were enrolled into study. Demographic data, disease activities, the drugs used, frequency of infection, and sickle cell-related crisis before and after rheumatic disease diagnosis-treatment of the patients were examined.

**Results:**

Inflammatory rheumatic disease was detected in 14 of 28 sickle cell patients evaluated in the rheumatology department for musculoskeletal complaints. Twelve of the patients were female and 2 were male. The median duration of rheumatic disease was 27 months (16.5). Eight of these patients had rheumatoid arthritis, 1 had ankylosing spondylitis, reactive arthritis, gout, connective tissue disease, undifferentiated monoarthritis, and 1 patient had undifferentiated oligoarthritis. For rheumatic disease, 11 (78.6%) of the patients were using steroids, 8 (57.1%) hydroxychloroquine, 4 (28.6%) methotrexate and sulfasalazine, 2 (14.3%) leflunomide, 1 (7.1%) anti-TNF (etanercept), and 1 allopurinol and colchicine. The frequency of SCD-related crisis and annual serious infections before and after rheumatic disease treatment were similar (p = 0.31).

**Conclusion:**

The clinical manifestations of inflammatory arthritis such as rheumatoid arthritis and sickle cell disease may overlap. The use of drugs such as steroids, methotrexate, or anti-TNF in sickle cell patients with rheumatic disease is the same as in patients without sickle cell disease. However, treatment should be individualized in patients with complications such as infection.

## 1. Introduction

Sickle cell disease (SCD) is a hematological disease that can be seen all over the world and it clinically progresses with attacks such as hemolytic crisis (HC) and vaso-occlusive crisis (VOC). The distortion of erythrocytes into a sickle shape in deoxygenated environment and development of vaso-occlusion and inflammation in the vessel wall are the main pathogenic mechanisms. SCD is an important cause of morbidity and mortality by causing chronic end-organ damage, in addition to its acute complications [1].

Musculoskeletal symptoms are common in sickle cell disease. Findings that may require orthopedic surgery, such as septic arthritis, avascular necrosis, and osteomyelitis, are important findings during SCD. In addition, extremity pain, arthralgia, myalgia, arthritis, and dactylitis can be seen [2].

On the other hand, inflammatory arthritis such as rheumatoid arthritis (RA) is common in the community, and this situation can create various difficulties in diagnosis and treatment in those especially with accompanying SCD. As the symptoms of inflammatory arthritis mimick VOC, diagnosis of inflammatory arthritis may be delayed in patients with SCD. Again, due to risk of triggering painful veno-occlusive crises in SCD, there may be difficulties while using drugs such as steroids recommended in the treatment of inflammatory arthritis [3]. In addition, the concomitant use of drugs such as hydroxyurea (HU) used in the treatment of SCD and disease modifying antirheumatic drugs (DMARDs) such as methotrexate (MTX) used in the treatment of RA have the potential to cause problems in terms of toxic side effects or drug interactions. Interestingly, SCD is known to be protective against malaria. However, diseases such as bacterial infections are an important cause of mortality in SCD, especially in children [4]. As for the patients who need to use biological drugs such as anti-TNF agents for rheumatic disease, treatment may be delayed due to the risk of infection.

In this study, we presented the clinical features and treatments of our patients with SCD who were followed up with a diagnosis of inflammatory rheumatic disease such as RA. Our main aim is to show whether the treatment of inflammatory arthritis in SCD is different and the secondary aim is to discuss the complications related to SCD that may develop with these treatments.

## 2. Method and patients

The study was conducted in an area where SCD is relatively common [5]. Patients aged 18 years and over who were followed up by the Hematology department with the diagnosis of SCD between January 2018 and June 2020 and who were admitted to the Rheumatology outpatient clinic due to musculoskeletal complaints were retrospectively screened. Besides demographic and clinical characteristics of the patients, laboratory findings and treatments they received were also evaluated. Patients, who were diagnosed, followed up and treated for inflammatory arthritis or rheumatic disease, were included in the study. The diagnosis of RA was made according to the 2010 ACR-EULAR criteria, and the diagnosis of ankylosing spondylitis (AS) was made according to the 1984 Modified New-York criteria [6, 7]. Patients with other inflammatory arthritis were included in the study if the disease was chronic (more than 6 weeks) or relapsing frequently and if they were irrelevant of an SCD veno-occlusive crisis. Patients who were not diagnosed with a rheumatic disease and who had SCD-related findings such as arthralgia and synovitis associated with attacks (VOC) such as musculoskeletal complaints were not included in the study. Patients with genetically confirmed SCD by the hematologist (GI) were included in the study, and patients with non-SCD hemoglobinopathy were excluded from the study. High Performance Liquid Chromatography (HPLC), a type of hemoglobin electrophoresis method was used to diagnose the patients. Rheumatic diseases, laboratory parameters, comorbidities, complications related to SCD, drugs used and doses, average annual number of SCD crises in the last 10 years, and average annual number of serious infections were recorded. Serious infection was defined as an infection that resulted in death, required hospitalization, or required parenteral antibiotic therapy for more than 2 weeks.

SPSS v. 22 for Windows program was used for statistical analysis. The conformity of the data to normal distribution was checked with the Shapiro–Wilk analysis test. The distribution of measurable (quantitative) data was expressed as mean ± standard deviation. Variables with nonnormal distribution were expressed as median and minimum-maximum, or interquartile range (IQR) values. The difference between the patient and control groups that did not fit the normal distribution was analyzed with the Mann–Whitney U test. The distribution of categorical data was given as % (percentages). p-values below 0.05 were considered statistically significant in all tests.

Ethics committee approval of the study was obtained from the ethics committee of Hatay Mustafa Kemal University Faculty of Medicine.

## 3. Results

In total, inflammatory rheumatic disease was found in 14 patients out of 28 patients with hemoglobinopathy who were examined rheumatologically for musculoskeletal complaints, after excluding patients with non-SCD hemoglobinopathies (thalassemia intermedia 3 patients) and those with SCD diagnosis whose musculoskeletal complaints were found to be associated with SCD. Twelve of the patients were female and 2 were male (85.7% female). The median age of the patients was 41 (16.75) years, and the median age at diagnosis was 38.5 (15.75) years. The median duration of rheumatic disease was 27 months (16.5). Eight of these patients (57.1%) had RA, 1 had AS, 1 had reactive arthritis, 1 had gout, 1 had connective tissue disease, 1 had undifferentiated monoarthritis, and 1 patient had undifferentiated oligoarthritis. Of the patients, 50% had at least one comorbidity. Rheumatoid factor (RF) was positive in 6 patients (42.9%), anticyclic citrulline peptide (CCP) was positive in 4 patients (28.6%), and antinuclear antibody (ANA) was positive in 2 patients (14.3%) (Table 1).

In the patient with gout (patient no: 12), uric acid (UA) level was 11.7 (3–7) mg/dL. In the previous laboratory findings of the patient, it was determined that he had chronic hyperuricemia and had acute monoarthritis attacks for 7–10 days in the first metatarsophalangeal joints, sometimes in the metatarsals and ankles for the last 3 years, and he was diagnosed with gout 3 years ago. The attacks he had about 2 years before gout diagnosis were estimated to be SCD crises, and there was a delay of approximately 2 years in the diagnosis of gout. The patient’s last gout attack was 5 months ago and UA level at the last visit was 5.8 mg/dL. The patient with AS (patient no: 9) had radiographic bilateral grade 2 sacroiliitis and the patient’s HLA B27 was negative. Patients with undifferentiated monoarthritis and undifferentiated oligoarthritis did not meet the ASAS (Assesment of SpondyloArthritis international Society) criteria for peripheral spondyloarttritis (Table 1).

Among the musculoskeletal complications associated with SCD, osteoporosis was present in 10 patients (71.4%) and avascular necrosis (AVN) was present in 5 patients (35.7). AVN had developed before rheumatic diagnosis and treatment in all patients. Other SCD-related events were autosplenectomy in 8 (57.1%) patients, cholelithiasis and proteinuria in 5 (35.7%), cholecystectomy in 4 (28.6%), acute chest in 3 (21.4%), pulmonary hypertension (PHT) in 2 (14.3%), and cerebrovascular accident (CVA) in 1 (7.1%) patient. Demographic data of the patients, laboratory findings related to rheumatic disease, comorbidities and complications related to SCD are shown in Table 1.

Considering all the treatments they received for rheumatic disease, 11 (78.6%) patients were treated with steroids, 8 (57.1%) patients with hydroxychloroquine (HCQ), 4 (28.6%) patients with MTX, 4 (28.6%) patients with sulfasalazine (SLZ), 2 (14.3%) patients with leflunomide (LEF), and 1 (7.1%) patient with anti-TNF (etanercept). The patient diagnosed with gout (patient no: 12) was on colchicine and allopurinol treatment, and had a history of short-term steroid use during a gout attack. The patient with AS (patient no: 9) was in remission and did not use DMARDs or biological drugs and was followed with nonsteroidal antiinflammatory (NSAI) drugs. The treatments received by the patients for SCD and rheumatic disease are shown in Table 2. The number of patients using HU was 11 (78.6%). Along with HU, 3 patients used MTX, 2 patients LEF, 4 patients SLZ, 6 patients HCQ, and 1 patient allopurinol.

Of 8 SCD patients who were diagnosed with RA, 7 were female and 1 was male. Six of these patients were seropositive and 2 were seronegative. All RA patients were using DMARDs and/or steroids. The prednisolone dose of the patients using steroids was <7.5 mg/day. DMARDs used for RA were MTX (7.5–15 mg/week), LEF (10–20 mg/day), SLZ (1–2 g/day) and HCQ (200–400 mg/day). One RA patient (patient no: 4) had a history of anti-TNF (etanercept) use. The same patient was using all the RA drugs intermittently and irregularly, due to a history of frequent infections. In the other patients with RA, drug use was regular. The frequency of SCD-related crisis before and after rheumatic diagnosis and treatment was similar (p = 0.31). Again, the annual frequency of serious infections before and after rheumatic diagnosis and treatment was similar (p = 0.31). (Tables 2 and 3).

## 4. Discussion

In this study, we found that conventional DMARDs and steroids can be used regularly in our adult SCD patients with inflammatory rheumatic disease. All patients with a diagnosis of RA used conventional (c) DMARDs (MTX, LEF, SLZ, and HCQ) and steroids. A patient with RA used anti-TNF (etanercept), a patient with AS used NSAIDs, a patient with gout used colchicine and allopurinol. The use of DMARDs did not constitute an obstacle for hydroxyurea used in the treatment of SCD. We did not find a significant increment in the annual number of SCD crisis and infection in our patients before and after rheumatic disease diagnosis.

During SCD, acute and transient arthritis can often be seen in association with VOC. This may make it difficult to determine the prevalence of inflammatory arthritis in SCD. Different results have been reported regarding the prevalence of chronic inflammatory arthritis, such as RA, in SCD. While the prevalence of RA in SCD was lower in older studies, in a more recent study, it was found that the prevalence of RA in SCD patients was similar to the prevalence of RA in the general population (0.5%–1%), and the prevalence of RA in SCD patients was found to be 0.94% [8, 9]. This situation may lead to delayed diagnosis of RA and more severe disease in areas with a high prevalence of SCD [10]. In the study by McFarlane et al., more periarticular osteopenia, erosive arthritis, and more severe disease index were found in patients with RA, and it was stated that the possible delay in diagnosis was higher in patients with both SCD and RA [9]. In our study, osteopenia was also common, but due to the small sample size, erosive arthritis or disease severity was not compared with RA patients without SCD. In our study, no significant delay in diagnosis was found, considering the age at diagnosis and mean disease duration of the patients. One reason for this may be that SCD patients are followed in a center that is highly experienced in hemoglobinopathy. In addition, there was no significant difference in the frequency of hospitalization and infection, before and after the diagnosis and treatment of RA in our SCD patients (Table 3).

Except for one of 8 patients with RA who participated in our study (Case 7), all the patients were female (87.5%). RF was positive in 75% and anti-CCP in 50% of our RA patients. Two of our patients (Cases 10 and 14) were seronegative. The seropositivity rate of our patients with RA was found to be 75%, which is consistent with the previous studies. Interestingly, seronegative RA patients in our study were younger than seropositive patients. Case 8 was the oldest patient in our study and was a 70-year-old female. The mortality rate is increased in SCD patients, and the life expectancy of patients in developed countries is in their 40s and 50s. Infections are an important cause of mortality in both children and adults. In addition, disease-related VOC and organ failure contribute to mortality [11]. Our patient (case 8) had approximately 6 years of RA disease duration and was positive for RF and anti-CCP. She used steroids, MTX, HCQ, and LEF for RA treatment and the patient was in remission. She had comorbid hypertension and atrial fibrillation, and also had a history of SCD-associated CVA, osteoporosis, AVN, cholelithiasis, autosplenectomy, and PHT. The mean annual number of infections of the patient before and after RA treatment was similar. While SCD was thought to be a childhood disease long ago and the number of patients who came to adulthood was thought to be lower, life expectancy has increased with the increase in the quality of care and more effective treatments over the years [12]. In our study, the 2nd and 3rd elderly patients were a 58-year-old female (case 11) and a 50-year-old female (case 4), and both patients had seropositive RA diagnosis. Case 11 was in remission, RA disease activity was stable with SLZ + HCQ, and low-dose steroid treatment. However, case 4 was a patient with erosive RA nonresponding to conventional DMARDs in need of biological (b) DMARDs and had a history of frequent hospitalizations due to infection and other complications of the disease. All the other patients were under 50 years of age. While mortality is higher in SCD patients due to acute complications in childhood, there is an increase in mortality due to chronic complications in adults [13]. Therefore, the risk of chronic inflammatory diseases such as RA may additionally contribute to mortality. Suppression of RA disease activity may be an effective factor in reducing the risk of morbidity and mortality in SCD patients.

However, in SCD patients, there is a risk of interaction between steroids and immunosuppressive agents such as MTX used in the treatment of diseases such as RA with the agents used in the treatment of SCD. In addition, immunosuppression caused by the drugs may increase the risk of infection.

In our study, 78.6% of our patients used steroids. The highest dose used in the acute arthritis period was 15 mg prednisolone and the daily maintenance dose was equivalent to <7.5 mg prednisolone. Three of our patients used only steroids in the treatment of arthritis. These were case 2; 34-year-old woman with undifferentiated monoarthritis, case 6; 37-year-old woman with undifferentiated oligoarthritis, and case 13; 47-year-old female diagnosed with reactive arthritis. Other patients who used steroids were also using antirheumatic drugs, in combination. In SCD, corticosteroids are not preferred by the clinicians because of the concern that they may increase rebound pain, avascular necrosis, and SCD-related crisis attacks. However, examples of the side effects of steroids in SCD are mostly reported in the form of case reports. In addition, many inflammatory cytokines such as tumor necrosis factor and interleukins are increased in SCD patients, especially in case of vaso-occlusive crisis, and occlusion, ischemia, and organ damage develop after intense inflammation. There are few controlled studies regarding the reduction of SCD crises by suppressing the inflammation with steroids in SCD patients [14]. However, SCD crises after intraarticular steroid injection have been described in the literature [15]. It has also been reported that the use of corticosteroids increases the frequency of hospitalization and may cause vaso-occlusive attacks [16, 17]. On the contrary, there are studies showing that steroids can be used in SCD patients and even that steroids can contribute positively to complications such as acute chest syndrome [18–21]. In our study, no steroid-induced SCD-related crisis was detected in any of the patients using steroids. Besides, all of our patients with a history of avascular necrosis that may be associated with both SCD and steroids, this complication occurred before the diagnosis of rheumatic disease and steroid use. In the literature, it is seen that especially patients who develop VOC use high-dose corticosteroids [17]. However, none of our patients used high-dose steroids. The absence of SCD-related crisis in our patients may be related to the use of low dose steroids in our patients. NSAI drugs can be used in SCD patients. However, they should be used with caution, by considering the potential risk factors such as gastrointestinal, renal, and cardiac side effects [22].

MTX is usually the drug of first choice in the treatment of RA patients. Apart from this, cDMARDs such as LEF, SLZ, and HCQ and bDMARDs such as anti-TNF are used in current RA treatment [23]. In our study, 4 patients (28.6%) with a diagnosis of RA were using MTX and 3 of these patients were receiving HU concurrently. The dose of MTX was 7.5–15 mg/week, and all patients were using folic acid. There are case reports in the literature regarding the use of MTX in patients with SCD with rheumatic disease, and it has been reported that there were patients who used the drug without any problems, as well as patients with an increased frequency of VOC when used with steroids [24, 25]. On the other hand, in a study examining the effects of MTX on VOC in SCD, it was reported that although MTX did not reduce the frequency and intensity of VOC crisis, it reduced chronic pain and had positive effects on quality of life in SCD patients [26]. In our study, we did not find an increase in the frequency of infection and VOC in patients using MTX, either with or without a history of steroid use. There are very limited data related to other cDMARDs such as LEF, SLZ, and HCQ used in the treatment of RA in SCD patients [25, 27–30]. Among our patients, 2 patients used LEF, 4 patients SLZ, and 8 patients used HCQ as monotherapy or in combination therapy. The doses of these agents are standard doses used in patients without SCD.

bDMARDs, such as anti-TNFs, are highly effective agents that can be used in many inflammatory arthropathies, especially RA. They are generally preferred in rheumatic patients with severe disease activity who do not respond to first-line treatments. However, undesirable side effects such as increased frequency of infection and activation of latent tuberculosis may be observed. Therefore, special attention should be paid to infection while using these drugs in SCD patients. However, there is no data regarding the increased risk of infection with bDMARDs in patients with SCD compared to those without SCD. There are case reports regarding the use of both anti-TNF agents and other biological drugs such as rituximab in SCD patients, and in general, the side-effect profile appears to be similar to that of patients without SCD [31–33]. We had a patient with SCD who used bDMARDs for RA, and the patient could not tolerate the drug due to frequent infections (case 4). However, the patient had a history of frequent hospitalizations due to serious infections before using anti-TNF agents.

Although our study was conducted at a site where SCD patients are relatively common, it was conducted in a single center. One of the limitations of the study is that the data of the patients were collected retrospectively. Again, the small number of patients restricts the generalizability of the data. However, considering the scarcity of studies on the coexistence of SCD and rheumatic diseases, the results obtained may be helpful in making the treatment decision of the patients.

As a result, clinical findings of SCD and inflammatory arthritis like RA may overlap, and diagnosis and treatment of rheumatic diseases may be delayed. The use of steroids, cDMARDs or bDMARDs in SCD patients with rheumatic disease is the same as in patients without SCD. These drugs should be used, by considering the risk of infection, and the treatment should be decided individually.

## Figures and Tables

**Table 1:** Demographic and clinical data of the patients.

Patient no.	Age/years, Sex	Rheumatic disease	Age/years at diagnosis of rheumatic disease	Duration of rheumatic disease/months	Laboratory findings	Concomitant disease	Sickle cell disease complications
1	38, F	Connective tissue	35	32	ANA +3	Absent	Osteoporosis, autosplenectomy
2	34, F	Undifferentiated monoarthritis	32	29	-	Absent	Osteoporosis, Avascular necrosis, autosplenectomy
3	42, F	RA	40	20	CCP >200	HT, Depression	Acute chest, osteoporosis, cholelithiasis, autosplenectomy, PHT, proteinuria
4	50, F	RA	38	136	RF= 59	HT	Acute chest, osteoporosis, AVN, cholelithiasis, autosplenectomy, proteinuria
5	47, F	RA	44	28	RF= 78, CCP= 178	HT	AVN, osteoporosis, proteinuria
6	37, F	Undifferentiated oligoarthritis	35	24	RF= 52	Absent	Cholelithiasis, autosplenectomy
7	18, M	RA	16	18	RF= 720	Absent	AVN
8	70, F	RA	64	96	RF= 92 CCP>200	HT, atrial fibrillation	CVA, Osteoporosis, AVN, cholelithiasis, autosplenectomy, PHT
9	22, F	AS	18	48	X-ray	Absent	-
10	20, F	RA	17	31	ANA+3	Absent	Acute chest, autosplenectomy
11	58, F	RA	56	20	RF= 504 CCP>200	Absent	Osteoporosis
12	47, M	Gout	44	26	UA= 11.7	CRF	Osteoporosis, autosplenectomy, proteinuria
13	45, F	Reactive arthritis	45	1	-	Absent	Osteoporosis
14	40, F	RA	39	12	-	Absent	Osteoporosis

Abbreviations: RA: rheumatoid arthritis, AS: Ankylosing spondylitis, RF: rheumatoid factor IU (0–15), CCP: anti-cyclic citrulline peptide mg/dL (0–10), ANA: antinuclear antibody, UA: uric acid mg/dL, HT: hypertension, CRF: chronic renal failure, PHT: pulmonary hypertension, AVN: avascular necrosis, CVA: cerebrovascular accident

**Table 2:** Drugs used by the patients and complications.

Patient no.	Treatment of rheumatic disease	Treatment of hematological disease	Serious infection/year before rheumatic disease treatment	Serious infection/year after rheumatic disease treatment	Sickle cell crisis/year before rheumatic disease treatment	Sickle cell crisis/year after rheumatic disease treatment
1	HCQ	HU, exchange	1	0	6	5
2	Steroid	HU, Folic acid, ASA	0	0	3	3
3	MTX, HCQ, steroid	Fe chelation, folic acid, exchange	0	0	1	2
4	MTX, LEF, HCQ, etanercept	HU, Fe chelation, ASA, exchange	3	4	3	2
5	SLZ, HCQ, steroid	HU, Fe chelation, zinc	0	0	1	1
6	Steroid	HU, Fe chelation	0	0	1	1
7	SLZ, HCQ, steroid	HU, Fe chelation, exchange	0	0	1	1
8	Steroid, HCQ, MTX, LEF	HU, zinc, folic acid	2	1	1	1
9	NSAID	-	0	0	1	0
10	MTX, HCQ, steroid	HU, exchange	1	1	1	1
11	SLZ, HCQ, steroid	HU, exchange	1	1	1	1
12	Colchicine, allopurinol, steroid	HU, exchange, Folic acid, ASA, Fe chelation	2	1	3	3
13	Steroid	HU, zinc	0	0	1	0
14	SLZ, steroid	HU, Folic acid, Zinc, exchange	0	0	1	1

Abbreviations: HCQ: hydroxychloroquine, MTX: methotrexate, SLZ: sulfasalazine, LEF: leflunomide HU: hydroxyurea, ASA: acetylsalicylic acid, Fe: iron; NSAID: Nonsteroidal antiinflammatory drug.

**Table 3:** Complications before and after diagnosis and treatment of rheumatic disease.

	Before rheumatologic diagnosis	After rheumatologic diagnosis	p
Frequency of serious infections median (IQR)	0 (1)	0 (1)	0.31
Frequency of sickle cell disease crisis, median (IQR)	1 (2)	1 (1)	0.31
	Before rheumatologic diagnosis	After rheumatologic diagnosis	p
Frequency of serious infections median (min–max)	0 (0–3)	0 (0–4)	0.31
Frequency of sickle cell disease crisis, median (min–max)	1 (1–6)	1 (0–5)	0.31
